# Diagnostic accuracy of the Xpert MTB/RIF assay for extrapulmonary and pulmonary tuberculosis when testing non-respiratory samples: a systematic review

**DOI:** 10.1186/s12879-014-0709-7

**Published:** 2014-12-31

**Authors:** Laura Maynard-Smith, Natasha Larke, Jurgens A Peters, Stephen D Lawn

**Affiliations:** Department of Clinical Research, Faculty of Infectious and Tropical Diseases, London School of Hygiene and Tropical Medicine, Keppel Street, London, WC1E 7HT UK; MRC Tropical Epidemiology Group, Faculty of Epidemiology and Public Health, London School of Hygiene and Tropical Medicine, London, UK; The Desmond Tutu HIV Centre, Institute for Infectious Disease and Molecular Medicine, Faculty of Health Sciences, University of Cape Town, Cape Town, South Africa

**Keywords:** Tuberculosis, Diagnosis, Extrapulmonary, Xpert MTB/RIF, GeneXpert, Diagnostic accuracy, Systematic review, Sensitivity, Specificity

## Abstract

**Background:**

Although the evidence base regarding the use of the Xpert MTB/RIF assay for diagnosis of pulmonary tuberculosis (TB) when testing respiratory samples is well established, the evidence base for its diagnostic accuracy for extrapulmonary and sputum-scarce pulmonary TB when testing non-respiratory samples is less clearly defined.

**Methods:**

A systematic literature search of 7 electronic databases (Medline, EMBASE, ISI Web of Science, BIOSIS, Global Health Database, Scopus and Cochrane Database) was conducted to identify studies of the diagnostic accuracy of the Xpert assay when testing non-respiratory samples compared with a culture-based reference standard. Data were extracted and study quality was assessed using the QUADAS-2 tool. Sensitivities and specificities were calculated on a per-sample basis, stratified by sample type and smear microscopy status and summarised using forest plots. Pooled estimates were calculated for groups with sufficient data.

**Results:**

Twenty-seven studies with a total of 6,026 non-respiratory samples were included. Among the 23 studies comparing Xpert and culture done on the same samples, sensitivity was very heterogeneous with a median sensitivity of 0.83 (IQR, 0.68–0.94) whereas specificities were typically very high (median, 0.98; IQR, 0.89–1.00). The pooled summary estimates of sensitivity when testing smear-positive and smear-negative samples were 0.95 (95% CI 0.91–1.00) and 0.69 (95% CI 0.60-0.80), respectively. Pooled summary estimates of sensitivity varied substantially between sample types: lymph node tissue, 0.96 (95% CI, 0.72-0.99); tissue samples of all types, 0.88 (95% CI, 0.76–0.94); pleural fluid, 0.34 (95% CI, 0.24–0.44); gastric aspirates for diagnosis of sputum-scarce pulmonary TB, 0.78 (IQR, 0.68 – 0.85). Median sensitivities when testing cerebrospinal fluid and non-pleural serous fluid samples were 0.85 (IQR, 0.75-1.00) and 0.67 (IQR, 0.00-1.00), respectively.

**Conclusion:**

Xpert detects with high specificity the vast majority of EPTB cases with smear-positive non-respiratory samples and approximately two-thirds of those with smear-negative samples. Xpert is a useful rule-in diagnostic test for EPTB, especially when testing cerebrospinal fluid and tissue samples. In addition, it has a high sensitivity for detecting pulmonary TB when using gastric aspirate samples. These findings support recent WHO guidelines regarding the use of Xpert for TB diagnosis from non-respiratory samples.

**Electronic supplementary material:**

The online version of this article (doi:10.1186/s12879-014-0709-7) contains supplementary material, which is available to authorized users.

## Background

Extrapulmonary disease accounts for between 10% and 42% of cases of tuberculosis (TB), with the proportion being greater among children and those with immunodeficiency due to HIV co-infection [1],[2]. Following haematogenous spread of bacilli during primary pulmonary infection, extrapulmonary TB (EPTB) may later develop in any anatomic location [3]. Once the diagnosis has been considered, confirmation can be difficult, with sample collection from deep-seated tissues being challenging and the disease typically being paucibacillary. Culture on solid and/or liquid media is the gold standard for diagnosis. However, prolonged turnaround times and limited laboratory infrastructure in resource-limited settings undermine the utility of culture-based diagnosis in clinical practice. Histology is widely used for diagnosis where the technical expertise exists, but this is technically demanding, lacks specificity and is time-consuming [4]. Existing nucleic acid amplification tests (NAAT) used in high-resource settings have some utility although, to date, have been overly complex to use in most resource-limited settings.

The Xpert MTB/RIF (Xpert) assay (Cepheid Inc., Sunnyvale, CA, USA) is a cartridge-based, semi-automated, rapid molecular assay, which permits rapid TB diagnosis through detection of the DNA of *Mycobacterium tuberculosis* and simultaneous identification of a majority of the mutations that confer rifampicin resistance (which is highly predictive of multi-drug resistant TB [MDR-TB]) [5]. The evidence base regarding the use of Xpert on sputum samples in the diagnosis of pulmonary TB (PTB) in a wide range of high and low-burden settings is strong, with pooled summary estimates of sensitivity when testing smear positive and smear negative samples of 98% (95% CI, 97 – 99%) and 68% (95% CI, 59 – 75%), respectively, and a pooled specificity of 98% (95% CI, 97 – 99%) [6]. The assay has been CE-marked as compliant with European Union legislation, approved by the United States Federal Drug Administration (FDA) and was first endorsed by the World Health Organization (WHO) in 2010 for use as the initial diagnostic test in individuals suspected of having pulmonary MDR-TB or HIV-associated pulmonary TB [7]. While the assay is being widely implemented in many high burden countries for this purpose, uncertainty remains concerning its potential role in diagnosis of EPTB [8]. In addition, the utility of testing non-respiratory samples for the diagnosis of sputum-scarce PTB remains unclear, including gastric aspirate or stool samples in children unable to produce sputum.

An increasing number of studies have now examined the diagnostic accuracy of Xpert for TB diagnosis when testing a wide range of non-respiratory sample types and using a culture-based reference standard. In light of the need to develop/refine global and national guidelines on the use of Xpert when testing non-respiratory samples, this systematic review evaluated and synthesized the available data, taking into account the wide range of different types of samples studied.

## Methods

### Search strategy

A systematic search of Medline, EMBASE, ISI Web of Science, BIOSIS, Global Health Database, Scopus and Cochrane Database of Systematic Reviews was carried out on 6 November 2013 to identify journal articles and conference abstracts reporting data on the diagnostic accuracy of the Xpert MTB/RIF assay when testing non-respiratory samples. There were no restrictions on study design, patient demographics or whether TB case finding was active or passive. Reference lists of included studies and review articles were hand-searched. The study conformed to the PRISMA statement [9].

The search strategy is laid out in brief below:

(Free text search terms) “tubercul*” *OR* “TB” *OR* “MDR-TB” *OR* “XDR-TB” *OR* (Subject heading term) “Tuberculosis”

AND

(Free text search terms) “Xpert” *OR* “MTB/RIF Assay” *OR* “MTB/RIF” *OR* “GeneXpert” *OR* “Cepheid”.

Citations were exported into Endnote X7 and duplicates were removed. Two reviewers independently selected titles and abstracts against inclusion and exclusion criteria and the full text of any potentially relevant papers was reviewed.

### Eligibility criteria

Eligible studies reported on the diagnostic accuracy of Xpert MTB/RIF for TB when testing non-respiratory clinical samples (specifically excluding sputum, broncho-alveolar lavage fluid, nasopharyngeal aspirates and tracheal aspirates). Studies of both active and passive case finding were included, but those in which samples were selected based on pre-screening with another assay (eg smear microscopy) were excluded. Data reporting on the testing of gastric aspirate and stool samples for diagnosis of pulmonary TB were also included, as were studies examining the utility of testing urine samples to diagnose disseminated TB in HIV-infected patients. Acceptable reference standards were culture of *Mycobacterium tuberculosis* complex on solid and/or liquid media. Studies using other reference standards were excluded. The reference standard for rifampicin resistance was phenotypic drug susceptibility testing (DST).

There were no limits on the number of participants in a study or on the publication date. Studies were excluded if they were not in the English language, if they referred only to respiratory specimens, or if the results of respiratory and non-respiratory specimens were combined and could not be disaggregated. When authors reported studies with duplicated data, only the study with the larger sample size was included.

### Data extraction and data analysis

Data extraction, verification and study quality assessment were conducted by two of the authors using a standardised data extraction form. The retrieved data included country of study, age-group, gender and HIV status of patients, study setting, types and number of samples tested. The numbers of true positives (tp), true negatives (tn), false positives (fp) and false negatives (fn) were extracted on a *per sample* basis and entered into 2×2 tables. Sensitivity and specificity were calculated from these tables as tp/(tp + fn) and tn/(tn + fp), respectively, with 95% confidence intervals. Since some papers presented data from multiple sample types, multiple estimates were extracted from individual papers. No data were extracted on a per patient basis. In studies which used a composite reference standard, the culture results alone were used as the reference standard.

The primary analyses comprised sensitivity and specificity estimates calculated using all sample types from each paper, but these were disaggregated into two groups according to whether the culture-based reference testing was done on the same sample as the Xpert test or on a different sample. To investigate potential sources of heterogeneity, estimates of sensitivity and specificity were calculated separately for the following sample types: (i) lymph node biopsies and fine needle aspirates; (ii) all tissue types (including lymph node samples); (iii) cerebrospinal fluid (CSF); (iv) pleural fluid samples; and (v) non-pleural serous fluid samples. Additionally estimates were calculated separately for smear positive and smear negative samples and gastric aspirate samples as a method of diagnosing pulmonary tuberculosis. Data were presented on forest plots.

Median values and interquartile ranges (IQRs) for sensitivity and specificity were calculated for the primary analysis and all subanalyses. Pooled estimates of sensitivity and specificity for the analyses by sample type and smear status were calculated in Stata, using the bivariate random effects and random effects models, respectively [10]. In studies with small numbers of samples, zero events in cells of the 2×2 table, often precluded calculation of pooled estimates of sensitivity/specificity; and in these cases median values and IQRs were calculated. Data on the sensitivity and specificity of Xpert MTB/RIF for detection of rifampicin resistance (compared to phenotypic drug susceptibility) were also examined.

### Quality assessment

Quality assessment of studies was performed using the QUADAS-2 tool, examining bias and applicability of the studies with respect to four separate domains: patient selection, index test, reference standard and the flow and timing of patients through the study [11]. No overall summary score was calculated, but for each domain, any concern with regards to bias and applicability were qualified as ‘low’, ‘high’ or ‘unclear’. These results were then presented in graph and table form.

## Results

### Studies included

**Figure 1:**
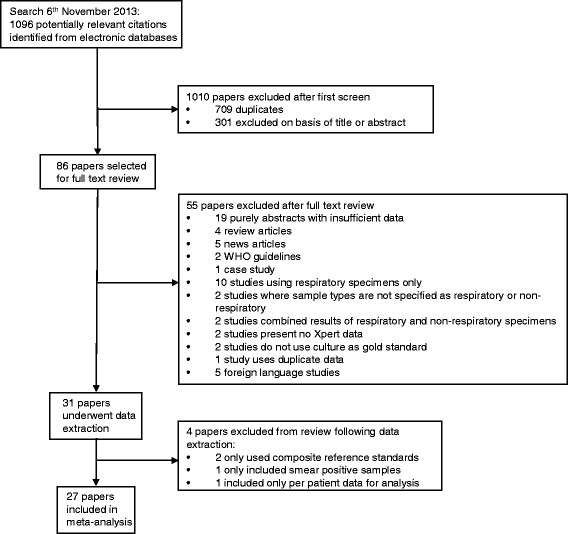
**Selection of studies reporting on the use of the Xpert MTB/RIF assay for diagnosis of tuberculosis from non-respiratory clinical samples.**

Of a total of 1096 citations, there were 387 unique potentially relevant citations identified and 301 of these were excluded after evaluation of the title and abstract (Figure 1). Of the remaining 86 papers selected for full text review, 55 papers were subsequently excluded, and 31 papers containing data on the diagnostic accuracy of Xpert MTB/RIF for TB when testing non-respiratory samples were reviewed in detail [12]-[38]. Of these, 27 studies published between January 2009 and November 2013 were eligible for inclusion in the qualitative and quantitative synthesis and reported on a total of 6026 non-respiratory samples (Table 1). Four additional studies were excluded on the basis of either using a composite reference standard (n = 2) [39],[40], including only samples known to be smear-positive (n = 1) [41] or providing per patient instead of per sample data (n = 1) [42] (Additional file 1: Table S1).

**Table 1:** **Characteristics of included studies**

First author	Year	Age (years)	% male	% HIV	Study setting	Sampling method*	Gold standard	Sample types (total n specimens analysed)	Sample used for gold standard	Composite reference standard included
**Studies included in meta-analysis**										
**Ablanedo-Terrazas**	2013	Adults >16 (Median 29, IQR 24–35.5)	88.2	100	Clinical	Prospective	Solid and/or liquid culture	Lymph node FNA, lymph node tissue biopsy (68)	As for Xpert	N/A
Mexico						Consecutive				
**Al-Ateah**	2012	NR	NR	NR	Laboratory	Prospective	Solid and/or liquid culture	CSF, Tissue/biopsy, Pleural fluid, Lymph node FNA, Pericardial, Peritoneal/ascites fluid, Synovial/articular fluid, Pus/abscess (67)	As for Xpert	N/A
Saudi Arabia						Consecutive				
**Armand**	2009	NR	NR	NR	Laboratory	Retrospective	Solid and/or liquid culture	Pleural fluid, Urine, Lymph node, Bone marrow, Pus/abscess\	As for Xpert	N/A
France						Convenience				
**Bates**	2011	Children <15, (Median 20 months, IQR 12 – 74 months)	NR	30.5	Clinical	Prospective	Liquid culture	Gastric aspirate (788)	As for Xpert	N/A
Zambia						Consecutive				
**Causse**	2009	Mean 45, Range 5 - 83	63.8	NR	Clinical	Prospective	Solid and/or liquid culture	CSF, Tissue/biopsy, Pleural fluid, Gastric aspirate, Pericardial, Peritoneal/ascitic, Synovial/articular, Pus/abscess (340)	As for Xpert	N/A
Spain						Consecutive				
**Deggim**	2010	NR	NR	NR	Laboratory	Prospective	Solid and/or liquid culture	CSF, Tissue/biopsy, Pleural fluid, Peritoneal/ascitic (7)	As for Xpert	N/A
Switzerland						Consecutive				
**Feasey**	2013	Adults only, 37	67	100	Clinical	Prospective Unspecified	Solid and/or liquid culture	Blood (104)	Sputum and/or blood	N/A
Malawi										
**Friedrich**	2011	NR	NR	NR	Clinical	Prospective Consecutive	Liquid culture	Pleural fluid (25)	Sputum and/or Abrams needle biopsy and/or pleural fluid	Culture *and/or* clinical presentation *plus* ADA level *plus* good response to anti-TB treatment
South Africa										
**Hanif**	2009	NR	NR	NR	Laboratory	Prospective	Solid and/or liquid culture	CSF, Pleural fluid, Urine, Gastric aspirate, Pus, Lymph node FNA (29)	As for Xpert	N/A
Kuwait						Unspecified				
**Hillemann**	2011	NR	NR	NR	Laboratory	Prospective	Solid and/or liquid culture	CSF, Tissue/biopsy, Pleural fluid, Urine, Gastric aspirate, Faeces (521)	As for Xpert	N/A
Germany						Consecutive				
**Ioannidis**	2011	NR	NR	NR	Laboratory	Prospective	Solid and/or liquid culture	CSF, Tissue/biopsy, Pleural fluid, Urine, Lymph node, Gastric aspirate, Pericardial, Synovial/articular, Pus/abscess (39)	As for Xpert	N/A
Greece						Unspecified				
**Lawn**	2012	Adults >18	39.3	100	Clinical	Retrospective	Liquid culture	Urine (168)	Sputum	N/A
South Africa						Consecutive				
**Ligthelm**	2011	4.2% under 5; 12.5% between 5 and 20; 83.3% over 20	41.6	18.8	Clinical	Prospective	Liquid culture	Lymph node FNA (48)	As for Xpert	Cytomorphology suggestive of TB with direct visualisation of the organism *and/or* bacterial culture
South Africa						Unspecified				
**Malbruny**	2011	Median 52	59.8	NR	Laboratory	Prospective	Solid and/or liquid culture	CSF, Tissue/biopsy, Pleural fluid, Urine, Lymph node, Gastric aspirate, Bone marrow, Peritoneal/ascitic fluid, Synovial/articular, Pus/abscess (122)	As for Xpert	N/A
France						Unspecified				
**Miller**	2011	NR	NR	NR	Laboratory	Retrospective	Solid and/or liquid culture	NR (23)	As for Xpert	N/A
USA						Unspecified				
**Moure**	2011	NR	NR	NR	Laboratory	Retrospective	Liquid culture	CSF, Tissue/biopsy, Pleural fluid, Gastric aspirate, Faeces, Lymph node, Pericardial, Peritoneal/ascitic fluid, Synovial/articular, Pus/abscess (149)	As for Xpert	N/A
Spain						Unspecified				
**Nhu (a)**	2013	Children under 16	NR	NR	Clinical	Retrospective	Liquid culture	Gastric aspirate (49)	As for Xpert	Confirmed case (acid-fast bacilli on smear microscopy or positive culture) *or* probable case (clinical symptoms consistent with TB, did not receive an alternative diagnosis and received TB treatment)
Vietnam						Consecutive				
**Nhu (b)**	2013	Adults >18	NR	20.8	Clinical	Prospective	Liquid culture	CSF (379)	As for Xpert	Culture *and/or* clinical symptoms *and/or* CSF criteria *and/or* cerebral imaging *and/or* evidence TB elsewhere
Vietnam						Consecutive				
**Nicol**	2013	Children <15 (Median 31 months, IQR 19 – 57 months)	NR	14.8	Clinical	Prospective	Liquid culture	Stool (115)	Sputum	N/A
South Africa						Consecutive				
**Peter**	2012	Median 35, IQR 28-38	41	100	Clinical	Prospective	Liquid culture	Urine (175)	Sputum and/or non-sputum	N/A
South Africa						Random				
**Porcel**	2013	Mean 50	58	0	Clinical	Prospective	Solid culture	Pleural fluid (67)	As for Xpert	Smear *and/or* culture of pleural fluid/sputum; *or* granuloma on pleural biopsy; *or* lymphocytic effusion with increased ADA; *and* resolution of effusion with TB treatment
Spain						Consecutive				
**Teo**	2011	NR	NR	NR	Laboratory	Unspecified	Solid and/or liquid culture	CSF, Urine, Pleural fluid, Pericardial fluid, Peritoneal/ascitic fluid, Tissue/biopsy, Pus/abscess (31)	As for Xpert	N/A
Singapore						Unspecified				
**Tortoli**	2012	NR	NR	NR	Laboratory	Retrospective	Solid and/or liquid culture	CSF, Tissue/biopsy, Urine, Pleural fluid, Gastric aspirate, Pus/abscess, Peritoneal, Synovial/articular, Pericardial (1493)	As for Xpert	Culture *and/or* radiological and/or histological signs suggesting TB and improvement after anti-TB treatment
Italy						Consecutive				
**Vadwai**	2011	Median 37, 8 months – 94 years	45.9	3	Clinical	Prospective	Solid and/or liquid culture	CSF, Tissue/biopsy, Pleural fluid, Lymph node FNA, Pericardial, Peritoneal/ascitic fluid, Synovial/articular, Pus/abscess (547)	As for Xpert	Confirmed (culture) *or* probable (clinical symptoms, radiological findings and/or histology/cytology suggestive of TB) *or* possible (only clinical symptoms and/or signs suggestive of TB)
India						Consecutive				
**Van Rie**	2013	Aged >18, Mean 25.8, Range 18 – 73	51	100	Clinical	Prospective	Liquid culture	Lymph node FNA (373)	As for Xpert	Culture *and/or* smear microscopy *and/or* cytology
South Africa						Consecutive				
**Zeka**	2011	Median 47.5	57.6	NR	Clinical	Prospective	Solid and/or liquid culture	Pleural fluid, Tissue/biopsy, Peritoneal/ascitic fluid, CSF, Pericardial fluid, Urine (176)	As for Xpert	Culture *and/or* clinical diagnosis (clinical, pathological and/or radiological findings)
Turkey						Unspecified				
**Zmak**	2013	NR	NR	NR	Laboratory	Prospective	Solid and/or liquid culture	Blood, CSF, Pleural fluid, Urine, Faeces, Pericardial fluid, Peritoneal/ascitic fluid, Tissue/biopsy, Skin swabs (241)	As for Xpert	N/A
Croatia						Unspecified				

*Sample selection: Study units selected prospectively, or retrospectively from existing samples; Consecutive, random or convenience sampling method. ‘Unspecified’ refers to studies where there was no clear indication how the study participants were chosen.

In the 27 studies included, the number of samples studied ranged between 7 and 1,476, with a median of 115 (IQR, 39 – 340) (Table 1). Ten studies were from Europe, eight from Africa, seven from Asia and one from both North and Central America. The estimated TB incidence rates in the countries where studies were performed ranged between 3.6/100,000 population/year (USA) to 1003/100,000 population/year (South Africa) in 2012 [1]. Most studies (20 of 27) were prospective, 6 were retrospective and in one this was unclear. Twelve studies tested samples of only one type, whereas the remaining fifteen studies reported on testing of multiple sample types. The proportion of samples which were culture positive in the studies ranged from 5 – 100% (Table 1).

Three studies enrolled only children, 5 included only adults, 3 included all age-groups and the remaining 16 studies did not specify participant ages. Patient gender was reported in only 11 studies, with males comprising between 39.3% and 88.2% of participants. Among the 11 studies which reported HIV status, the proportions who were positive ranged between 0% and 100%. Study settings were classified as ‘clinical’ if patients were recruited to the study (14 studies), and ‘laboratory’ if samples were selected for inclusion within the laboratory (13 studies).

### Methodological quality of included studies

The methodological quality of included studies was assessed using the QUADAS-2 checklist [11]. A considerable proportion of studies was considered to be at high risk of bias in the patient selection and flow and timing domains (41% and 15% respectively) (Additional file 2: Figure S1 and Additional file 3: Figure S2). Within the patient selection domain, studies were considered to be at high risk of bias if a case–control study design was used, or because the study excluded certain groups of patients. The main reason studies were considered at high risk of bias within the flow and timing domain, was because the same reference standard was not used for all patients, or because not all the samples included in the study had been accounted for in the analysis. In 13 of 27 (48%) studies, applicability was considered of high concern with regard to patient selection in the laboratory-based studies.

### Overall diagnostic accuracy of Xpert MTB/RIF

**Figure 2:**
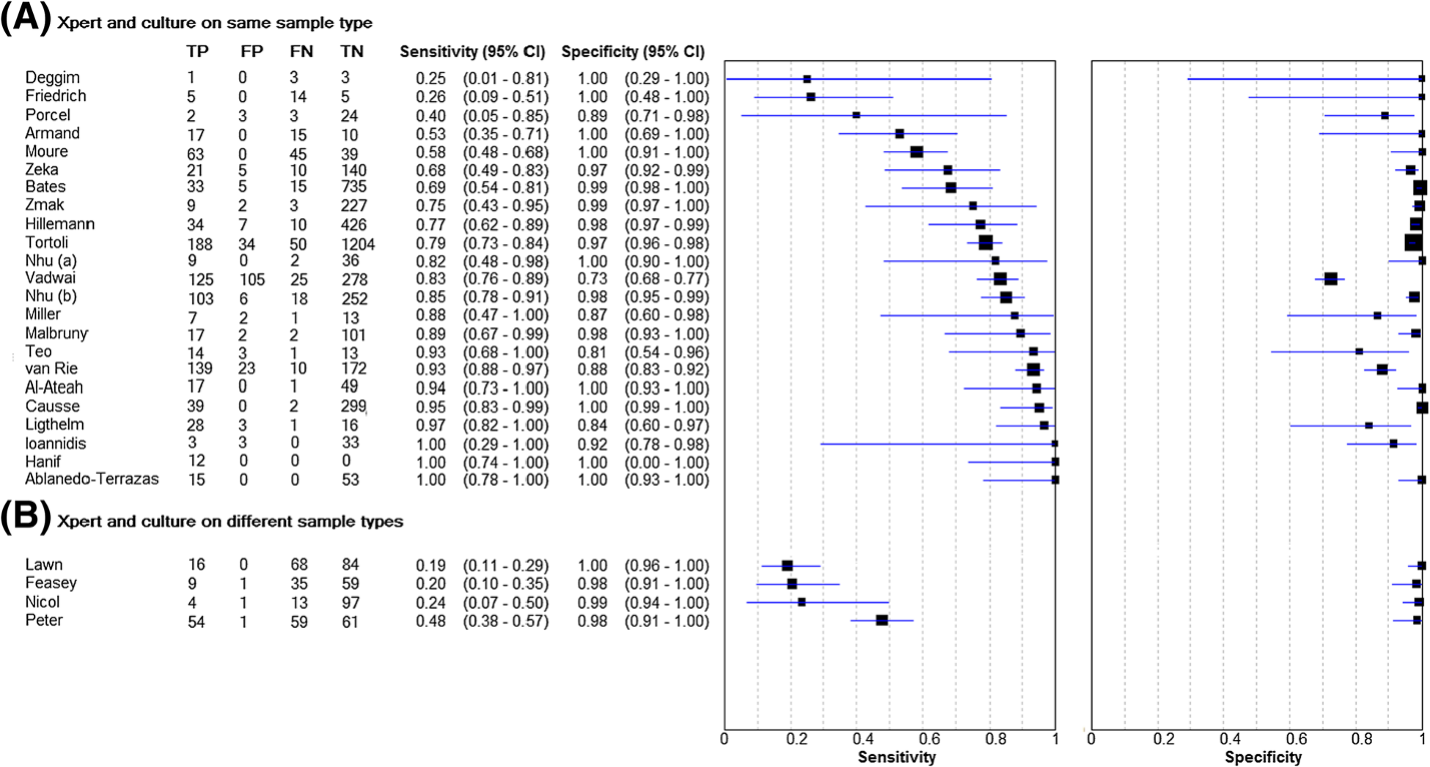
**Sensitivity and specificity of Xpert MTB/RIF assay (A) against culture on same sample type; (B) against culture on different sample types. (A)** Median sensitivity 0.83 (IQR 0.68 – 0.94), median specificity 0.98 (IQR 0.89 – 1.00); **(B)** Median sensitivity 0.22 (range 0.19 – 0.48), median specificity 0.99 (range 0.98 – 1.00)

Among the 23 studies comparing Xpert MTB/RIF with culture of the same sample or paired samples obtained from the same anatomic location of disease, the median sensitivity and specificity were 0.83 (IQR, 0.68 – 0.94; range, 0.25 – 1.00) and 0.98 (IQR, 0.89 - 1.00; range, 0.73 – 1.00), respectively (Figure 2). In contrast, among those studies (n = 4) in which the reference standard included culture of sample types which were different to the non-respiratory sample tested with Xpert (for example, sputum), the median sensitivity of Xpert was much lower (0.22; range 0.19 – 0.48) [18],[19],[23],[30],[31].

### Diagnostic accuracy in different sample types for EPTB

Disaggregated data were available by sample type for lymph node biopsies and fine needle aspirates, all tissue types (including lymph node samples), cerebrospinal fluid (CSF), pleural fluid and non-pleural serous fluid (pericardial, ascitic and synovial fluid).

**Figure 3:**
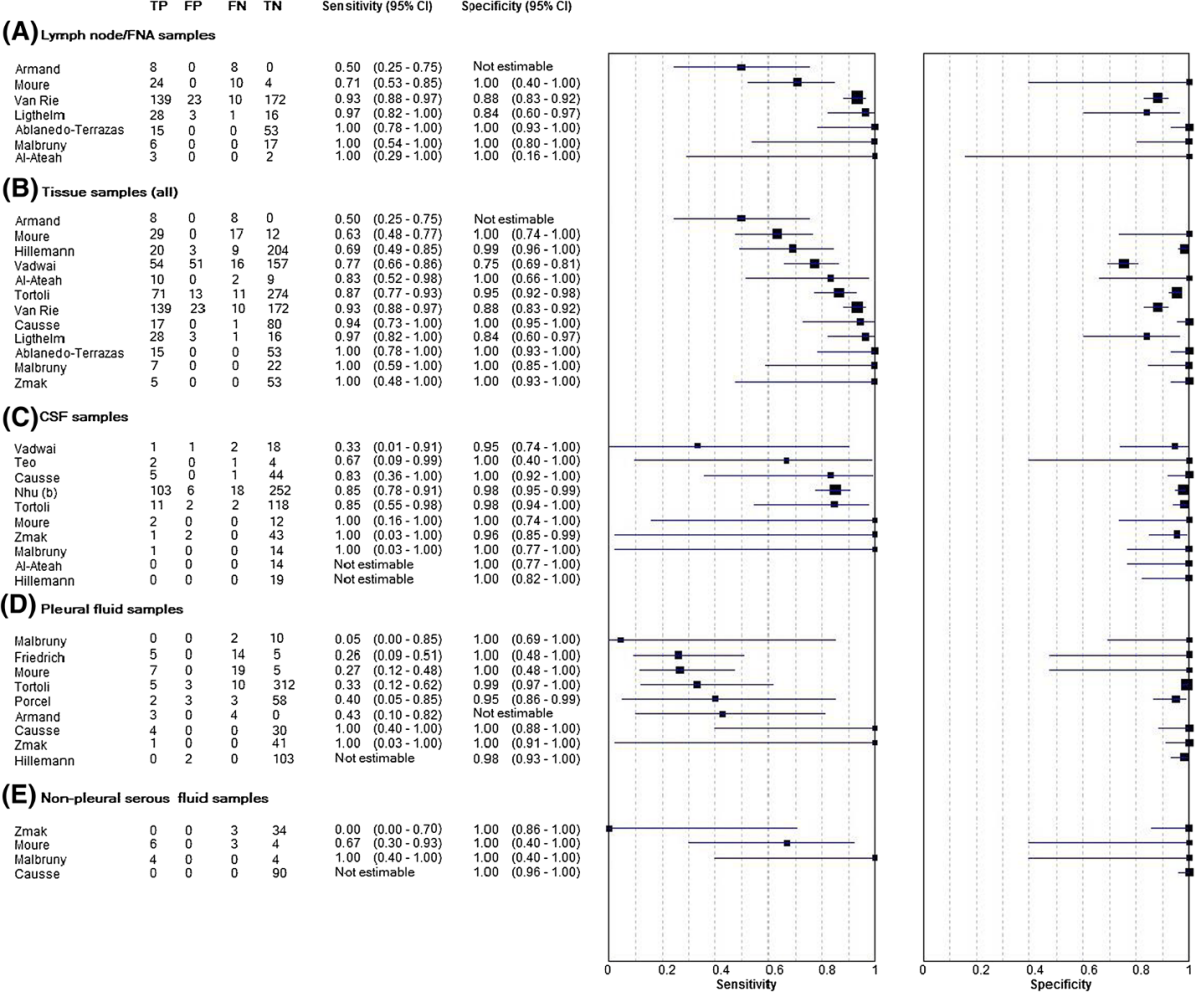
**Sensitivity and specificity of Xpert MTB/RIF assay in different sample types (A - E).**

The specificity of the Xpert assay was typically very high when used to test samples of all types, with median values of 1.00 for all sample types, including CSF, pleural fluid, non-pleural serous fluid, all tissue samples, lymph node sample and gastric aspirates. Pooled estimates of specificity could not be calculated for CSF or non-pleural serous fluids due to small numbers of samples in these studies, but pooled summary estimates for the remainder ranged from 0.93 to 0.99 (Table 2). The pooled specificity for lymph node tissue (0.93, 95% CI 0.70 - 0.99) was less than that for all tissue samples combined (0.98, 95% CI 0.87 – 0.99) (Table 2, Figure 3).

**Table 2:** **Sub-analyses of sensitivity and specificity by sample type and smear status**

Sample	Statistical model for pooled estimates	Number of studies	Sensitivity		Specificity	
			Pooled estimate (95% CI)	Median (IQR)	Pooled estimate (95% CI)	Median (IQR)
CSF	Cannot calculate	10		0.85 (0.75 – 1.00)		1.00 (0.98 – 1.00)
Pleural fluid	Bivariate	9	0.34 (0.24 – 0.44)	0.37 (0.27 – 0.72)	0.98 (0.96 – 0.99)	1.00 (0.98 – 1.00)
Non-pleural serous fluid	Cannot calculate	4		0.67 (Range 0.00 – 1.00)		1.00 (Range 1.00 – 1.00)
All tissue	Bivariate	12	0.88 (0.77 – 0.95)	0.90 (0.73 – 0.99)	0.98 (0.87 – 0.99)	1.00 (0.89 – 1.00)
Lymph node biopsy/FNA	Bivariate	7	0.96 (0.72 – 0.99)	0.97 (0.71 – 1.00)	0.93 (0.70 – 0.99)	1.00 (0.94 – 1.00)
Gastric aspirate	Bivariate	8	0.78 (0.69 – 0.86)	0.85 (0.74 – 1.00)	0.99 (0.98 – 0.99)	1.00 (0.99 – 1.00)
Smear positive	Random effects	9	0.95 (0.91 – 1.00)	1.00 (0.98 – 1.00)		
Smear negative	Random effects	10	0.69 (0.60 – 0.80)	0.69 (0.61 – 0.83)

In contrast to specificity, sensitivity for TB was very heterogeneous within and between all sample types. Median values for sensitivity ranged from 0.37 – 0.97. The highest pooled estimate was for lymph node tissue (0.96, 95% CI 0.72 – 0.99, n = 6), which was lower when all tissue samples were aggregated (0.88, 95% CI 0.77 – 0.95, n = 11). The lowest pooled sensitivity estimate was that for testing pleural fluid samples (0.34, 95% CI 0.24 – 0.44, n = 9) (Figure 3, Table 2).

### Diagnostic accuracy in gastric aspirate and stool samples for PTB

**Figure 4:**
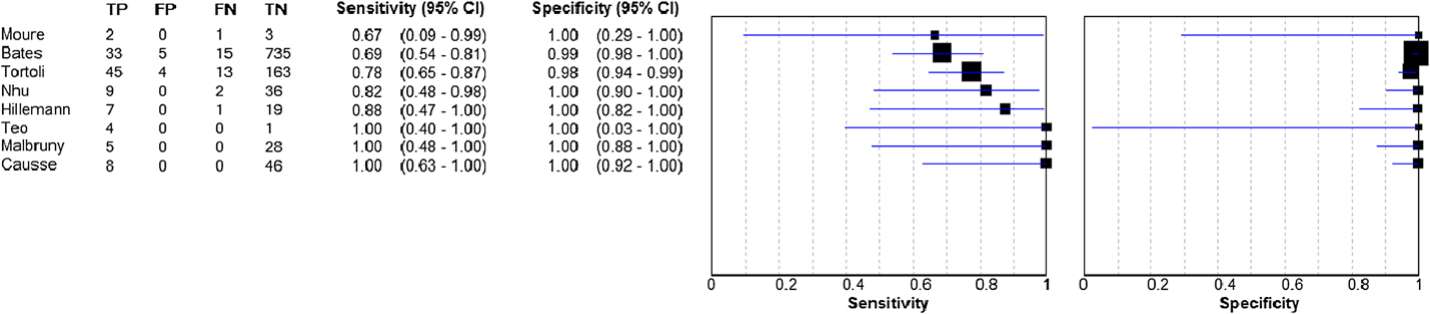
**Sensitivity and specificity of Xpert MTB/RIF assay against a culture reference standard on gastric aspirate samples.**

As shown with other sample types, the specificity of using Xpert to test gastric aspirates for diagnosis of pulmonary TB is extremely high (pooled estimate 0.99, 95% CI 0.98 – 0.99, range, 0.98 – 1.00), with a sensitivity pooled estimate of 0.78 (95% CI 0.69 – 0.86, range, 0.67 – 1.00) (Figure 4). Three studies reported on Xpert testing of stool samples compared to culture, but sample sizes were small at 2, 3, and 14 samples per study [21],[27],[38]. The sensitivity in the Hillemann et al. and Moure et al. papers were both 1.00, and the specificity in the Hillemann et al. and Zmak et al. papers were 0.92 and 1.00 respectively (data not shown on forest plot).

Two of the gastric aspirate studies were carried out only with children: Bates et al., sensitivity 0.69 (95% CI 0.54 - 0.81), specificity 0.99 [95% CI 0.98 - 1.00]; Nhu et al., sensitivity 0.82 (95% CI 0.48 - 0.98), specificity 1.00 (95% CI 0.90 – 1.00) [15],[29]. One further study compared stool samples to culture of sputum in children and found a sensitivity of 0.24 (95% CI 0.07 – 0.5) [30]. These results could not be pooled due to the different reference standards between the gastric aspirate studies and the latter study on stool samples. It is likely that the gastric aspirate samples in other studies were collected from children, but age-stratified data were not available and therefore could not be included on an analysis of Xpert MTB/RIF in children.

### Association between diagnostic accuracy and smear status and HIV status

**Figure 5:**
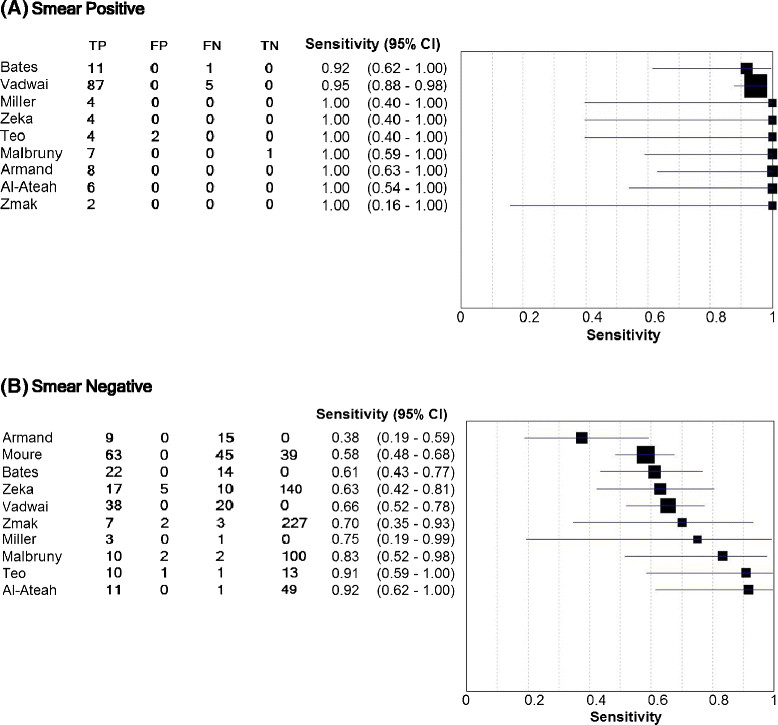
**Sensitivity of Xpert MTB/RIF assay in (A) smear positive and (B) smear negative samples.**

Sub-analysis of studies comparing Xpert and culture on smear positive and negative samples was conducted. The sensitivity when testing smear-positive samples (pooled estimate 0.95, 95% CI 0.91 – 1.00, range 0.92 – 1.00) far exceeded that observed when testing smear-negative samples (pooled estimate 0.69, 95% CI 0.60 – 0.80, range 0.38 – 0.92) (Figure 5, Table 2). Only one study provided specificity data for smear positive samples, so pooled estimates and comparisons between smear positive and negative samples were not made.

It was not possible to adequately assess the relationship between HIV status and diagnostic accuracy of Xpert MTB/RIF as only 2 studies provided suitable data. Bates *et al.* studied gastric aspirates from children and found that sensitivity did not differ significantly between HIV negative children (0.74, 95% CI 0.55-0.88) and HIV positive children (0.63, 95% CI 0.36-0.84) [15]. In contrast, Nicol *et al.* tested stool samples from children and found that the sensitivity in HIV positive children (0.80, 95% CI 0.38-0.96) was substantially higher than that observed in HIV negative children (0.33, 95% CI 0.14-0.61; p-value 0.08) [30].

### Variations in study methodology

Study methodology varied greatly across all 27 studies. Sixteen studies used a combination of solid and/or liquid culture as a reference standard, ten studies used only liquid culture and one study used only solid culture (Table 1). Sub-analyses of the pooled estimates for different sample types and smear status by solid or liquid culture were not possible, due to the large number of studies using either solid, or liquid culture, or both.

The methods of studies using CSF and pleural fluid samples were examined to try and establish differences in accuracy when using concentrated and unconcentrated samples. Frequently the methods were not described in sufficient detail (i.e. the volume of fluid used) to allow for sub-analyses on this basis (Additional file 4: Table S2).

### Detection of rifampicin resistance

The accuracy of Xpert MTB/RIF for detecting rifampicin resistance compared to phenotypic drug susceptibility testing (DST) was reported for non-respiratory samples in only 6 studies. The total number of samples testing positive for *M. tuberculosis* by Xpert MTB/RIF that were tested by phenotypic DST was 259 and included a variety of sample types (Table 3). However, the prevalence of rifampicin resistance by phenotypic DST in these studies was 0% for those carried out in Europe and sensitivity could not be estimated [21],[27],[38]. The sensitivity for rifampicin resistance was high in the remaining 3 studies from Saudi Arabia (1.00), India (0.98) and South Africa (1.00). Specificity was variable (range, 0.87-1.00).

**Table 3:** **Rifampicin resistance detection by Xpert compared to phenotypic drug susceptibility testing**

Study	Xpert positive samples tested for drug resistance (n)	Rifampicin resistance detection				Prevalence of rif resistance by DST	Sensitivity	Specificity
		TP	FP	FN	TN			
Al-Ateah	17	2	0	0	15	11.7%	1.00	1.00
Hillemann	26	0	1	0	25	0%	Not estimable	0.96
Moure	63	0	0	0	63	0%	Not estimable	1.00
Vadwai	125	39	5	1	80	31.2%	0.98	0.94
Zmak	12	0	0	0	12	0%	Not estimable	1.00
Lawn*	*16*	*1*	*2*	*0*	*13*	*6.3%*	*1.00*	*0.87*

*Comparison of urine Xpert MTB/RIF vs sputum phenotypic DST.

### Indeterminate test results and non-tuberculous mycobacteria

The proportion of Xpert MTB/RIF indeterminate results was reported by 14 studies, and the median was 1.4% (IQR, 0–4.1%; range 0–26.9%). A high proportion (26.9%) was reported by Feasey *et al.* when testing blood samples. Three studies reported data on non-tuberculous mycobacteria (NTM) identified by culture from 79 samples. Of these, 77 tested negative by Xpert and two had indeterminate results.

## Discussion

In this systematic review, we have carefully evaluated the literature on the diagnostic accuracy of the Xpert MTB/RIF assay when used to test non-respiratory samples and this synthesis supports its use in the diagnosis of both EPTB and sputum-scarce pulmonary TB. The specificity was very high across the majority of studies, highlighting its utility as a rule-in test for TB diagnosis that can be used to reliably inform the start of TB treatment when positive. In contrast, sensitivity was extremely heterogeneous, with much higher sensitivity being typically seen when testing lymph node samples, other tissue samples and cerebrospinal fluid as compared to the results of testing pleural fluid and other serous fluids. These findings strongly support the recently released WHO recommendations for the use of Xpert MTB/RIF for TB diagnosis by testing CSF and tissue samples [43].

Sensitivity ranged between 0.25 and 1.00 when Xpert and culture were both applied to the index non-respiratory samples. A key source of heterogeneity was the smear-status of samples as previously observed described when testing sputum samples for pulmonary TB diagnosis [6]. Xpert reliably detected the vast majority of non-respiratory samples testing smear-positive, culture-positive for *M. tuberculosis* but approximately only two-thirds of smear-negative samples. The type of non-respiratory sample tested was another key variable associated with the heterogeneity in sensitivity. Not only were pooled and median sensitivities typically far higher when testing lymph node or other tissue samples and cerebrospinal fluid compared to those observed when testing pleural or other non-pleural serous fluids, but high sensitivity was also observed when testing gastric aspirate samples highlighting an exciting new opportunity in the diagnosis of sputum-scarce pulmonary TB, which is potentially particularly useful in children.

Other potential sources of heterogeneity in sensitivity include small sample sizes, differences in study design, patient selection, patient age (adult versus child) and sample processing methodology. Despite this, high specificity suggests that the Xpert assay could play a useful role as a rapid rule-in test for diagnosis of EPTB, particularly when used to test lymph node samples, tissue biopsies or CSF samples. Limited sensitivity would, however, preclude use of the assay to rule-out EPTB, especially when samples have also been demonstrated to be smear-negative. Collectively, these data suggest that this assay represents an important advance in the diagnosis of EPTB and sputum-scarce pulmonary TB using non-respiratory samples. However, further well-designed, large studies are still needed to definitively characterise the accuracy and impact of this assay, optimum means of obtaining and processing samples.

A systematic review recently published by Denkinger et al., also carefully examined the use of the Xpert assay in extrapulmonary TB [44]. They searched four databases and included 18 papers with a total of 4461 samples, compared to the 6026 samples from 27 studies included in this review. The review by Denkinger et al. included studies with more than 10 samples of either lymph node tissue, cerebrospinal fluid or pleural fluid, and did not examine the use of non-respiratory samples for diagnosing sputum-scarce pulmonary TB. They examined sensitivity and specificity against both culture and composite reference standards, and for each sample type found that sensitivity increased and specificity decreased against a composite reference standard. We did not compare the findings of Xpert with a composite reference standard since it was evident that this was a further source of substantial heterogeneity since composite reference standards varied so much between studies.

The high pooled estimate of sensitivity we found in smear positive samples (0.96, 95% CI 0.91 – 1.00) is reflected in the findings by Denkinger et al. (0.97, 95% CI 0.96 – 0.99). The latter did not analyse smear negative samples, which our study found to have a much lower pooled sensitivity estimate (0.69, 95% CI 0.60 – 0.80). The very strong association between sample smear status and the sensitivity of Xpert MTB/RIF is entirely consistent with the findings observed when testing sputum samples, reflecting the mycobacterial load present [6]. In vitro experiments in which sputum samples have been spiked with *M. tuberculosis* have found that the assay retains 95% reliability for detection of the organism in sputum samples spiked with as few as 131 colony forming units per millilitre [45]. Comparable data are not known for non-respiratory samples types but the threshold for detection may differ according to the degree of polymerase chain reaction (PCR) inhibition associated with various sample types. The Xpert assay will provide an invaluable aid to diagnosis of EPTB in which smears are negative, especially in settings with otherwise limited laboratory capacity. Moreover, for smear-positive samples, Xpert provides a rapid and reliable means of differentiating between *Mycobacterium tuberculosis* complex and non-tuberculous mycobacteria as well as providing a rapid screening for rifampicin-resistance mutations.

We found a higher pooled sensitivity when testing lymph node samples than Denkinger et al. who included data from a larger number of studies (0.96, 95% CI 0.72 – 0.99, n = 7 compared with 0.83, 95% CI 0.71 – 0.91, n = 13) [44]. Several of the studies that we included in the tissue sample group did not describe from which anatomical location the biopsies originated. It is likely that many of the additional data included in that group are also lymph node samples. This may explain why the pooled estimate for all tissue samples in our study is more comparable with the Denkinger et al. findings for lymph node tissue (0.88, 95% CI 0.77 – 0.95, n = 12) [44]. Two studies included in our review from South Africa used samples obtained from lymph nodes via fine needle aspiration and reported high sensitivities of 0.93 (Ligthelm *et al.*) [24] and 0.97 (van Rie *et al.*) [36], with most of these patients having HIV-coinfection. The very high sensitivities reported are likely to reflect extremely high mycobacterial burden within lymph nodes of these patients and, consistent with this, van Rie *et al.* found that sensitivity was inversely related to blood CD4 cell counts. Ablanedo-Terrazas *et al.* tested both fine needle aspirates and excision biopsies in HIV positive patients, and found sensitivities of 1.00 regardless of the level of immunosuppression [12]. Denkinger et al. examined whether there was a difference in sensitivity between studies with >10% HIV positive patients and <10%, and found that accuracy rates did not differ [44]. This disparity demonstrates that more work needs to be done to differentiate the impact of HIV on the accuracy of Xpert in tissue samples. As well as HIV-infected patients, children may also benefit from improved diagnosis on lymph node tissue, as up to 50% with extra-thoracic TB have been shown to have cervical lymphadenitis [46].

TB meningitis (TBM) accounts for approximately 1% of TB disease, but carries a very high mortality risk of between 20% and 50% [47]. Prognosis worsens with later presentation, which may be exacerbated by prolonged time to diagnosis and treatment initiation. The sensitivity of smear microscopy in some cases can be as low as 20% of culture-confirmed disease, increasing with larger volumes and longer examination times of CSF [48]. Although culture provides considerably increased sensitivity, prolonged turnaround times mean that initiation of treatment for TBM is frequently empirical. The high median sensitivity of Xpert when testing CSF (0.85, IQR 0.75 – 1.00) is therefore an important finding, complemented by similar findings by Denkinger et al. (pooled sensitivity 0.81, 95% CI 0.59 – 0.92) [44]. One study by Patel *et al*, was excluded from our data synthesis on the basis of its use of a composite reference standard rather than culture alone [39]. However, a key observation was that testing the pellet from 3 ml of centrifuged CSF provided a sensitivity of 0.65 (95% CI 0.47-0.80) compared to a sensitivity of 0.26 (95% CI 0.17 – 0.37; p <0.001) when just 1 ml of uncentrifuged CSF was tested. Denkinger et al. compared concentrated and unconcentrated samples in CSF samples from several studies and also found that sensitivity and specificity were higher in the former group (84.2% vs 51.3%, 98% vs 94.6% respectively) [44]. The study protocols for all the studies included in this review have been summarised, but there were not sufficient data available to allow for further sub-analyses (Additional file 4: Table S2).

The lower sensitivities in pleural and non-pleural serous fluid likely reflect the low bacillary burden in these samples. It is known that analysis of pleural biopsies rather than pleural fluid increases microbiological diagnosis as well as allowing histological evaluation [49]. The study by Christopher *et al.*, which was excluded on the basis of use of composite reference standards, compared the Xpert assay on both pleural fluid and pleural biopsies [40]. However, this study found no benefit from testing pleural biopsy samples with Xpert. It was frequently unclear from the studies of pleural disease in this review what volumes of pleural fluid were used. As a result of this, sub-analyses on the effect of centrifugation were not carried out as the study methodologies for comparison could not be clearly defined (Additional file 4: Table S2). Studies in the Denkinger et al. review were found to have higher sensitivities where the proportion of HIV patients was <10%, and where a concentration step was used, but the paper comments that the confidence intervals are wide and overlapping. Further studies need to define optimum sample volumes for all sterile fluids, processing methodologies and the yield from pleural biopsies.

Missed and delayed diagnosis of TB in children is common, due to non-specific presentation, paucibacillary disease and the difficulty of expectorating sputum [50]. Two studies specifically sought to test gastric aspirates in children suspected of PTB [15],[29], where several other studies incorporated gastric aspirates into a wider study on all sample types with a mixed population. It is likely that the samples from the latter group were also predominantly children. The pooled sensitivity of Xpert on gastric aspirate samples was 0.78 (95% CI 0.68 – 0.85) compared to 0.25 for smear microscopy in the study by Bates et al. [15]. Stool samples are another means of testing for pulmonary disease in children, but the sample numbers in the studies included in this review comparing Xpert to culture on stool are very small. Nicol *et al*. compared stool samples to culture of sputum and found a sensitivity of 0.24 (95% CI 0.07 – 0.5) [30]. The paucity of age-stratified data in the studies in this review precluded any specific analysis on the use of Xpert MTB/RIF assay in children compared to adults.

Two further studies assessed the utility of Xpert in detecting disseminated disease from urine in HIV-infected out-patients and in-patients, using sputum culture as the reference standard [23],[31]. The sensitivity observed among selected sick medical hospital in-patients (0.48; 95% CI 0.38 – 0.57) [31] was substantially higher than that observed among unselected ambulatory out-patients undergoing active screening regardless of symptoms prior to starting antiretroviral therapy (0.19; 95% CI 0.11 – 0.29) [23]. There was a strong inverse association between Xpert sensitivity and blood CD4 cell count; sensitivity increased to 0.60 in in-patients with CD4 count <100 cells/ml [31]. Frequently, such patients are sputum-scarce, preventing the use of sputum smear microscopy, and therefore a rapid test with this sensitivity is critical to those patients who may have high mortality risk while waiting for culture results. Moreover, in combination with another urine rapid test (Determine TB-LAM), a more recent study has shown that sensitivity in this group can be increased further to 0.85 (95% CI 0.75-0.92) [51]. These results demonstrate that urine-based diagnosis may be particularly useful for routine investigation among HIV-infected medical in-patients.

A paucity of data precludes adequate evaluation of the accuracy of the Xpert assay for detecting rifampicin resistance from non-respiratory samples. The limited data available are similar to those observed when testing sputum samples with high sensitivity but sub-optimal specificity in some studies. However, it should be noted that all these studies were conducted prior to the launch of the modified G4 version of the assay cartridges for which evidence thus far shows much improved specificity in sputum [52].

Particular strengths of this review include the analysis of non-respiratory samples for diagnosis of PTB, the comprehensive search strategy and comparison of data to a uniform rigorous reference standard. Only five of the original 387 potentially relevant citations from the search needed to be excluded on the basis of language, which is not expected to have significantly altered the result. Stratification of data by sample type revealed the comparative utility of the assay for different forms of EPTB, and complements the meta-analysis by Denkinger et al. [44]. The findings of the review are limited by differences between studies including study design, clinical populations and patient selection, small sample sizes and non-standardized sample processing. All of these factors may have contributed to the marked degree of heterogeneity observed in the estimates of assay sensitivity. Insufficient data were available to adequately examine certain key variables such as age (adults vs children) and HIV status.

An important unresolved issue regarding the use of Xpert assay to test non-respiratory samples is the differing processing requirements for each sample type. Technical studies on optimum processing methods would enable the highest rates in sensitivity to be achieved, as well as lowering indeterminate rates. Adding in processing steps will mean that use of the assay will likely be restricted to secondary and tertiary care settings potentially limiting the impact of this technology. Larger scale implementation and cost-effectiveness studies should be carried out to identify the true benefits over culture in the time to treatment initiation and subsequent benefits in clinical outcomes. Further studies which allow stratification of data by HIV status or level of immunosuppression may inform how the assay might best be used in active case finding, particularly in areas with high rates of HIV, either alone or in combination with other diagnostic modalities.

## Conclusions

The findings of this review confirm that the Xpert MTB/RIF assay is an important advance in the diagnosis of EPTB and support the WHO guidelines issued by WHO in 2014 [43]. Principally, Xpert MTB/RIF is likely to be of greatest utility when testing CSF and lymph node or tissue samples, and differentiating tuberculous from non-tuberculous mycobacteria in smear positive samples of any type. In addition, there is clearly a role for testing gastric aspirate samples in diagnosis of sputum-scarce PTB. Further studies are needed to expand the evidence base for use of this assay for diagnosis of EPTB and PTB with different forms of non-respiratory samples in a wide range of clinical populations. However, the present data provide support for current implementation of this assay for EPTB diagnosis.

## Completing interests

The authors declare that they have no competing interests.

## Additional files

## Electronic supplementary material

Additional file 1: Characteristics of excluded studies. (DOCX 14 KB)

Additional file 2: Figure S1.: Number and percentage of studies within each domain of high, unclear or low risk of bias and concern of applicability, using the QUADAS-2 tool. (PDF 7 KB)

Additional file 3: Figure S2.: Scoring of each study for methodological quality for bias and applicability, using the QUADAS-2 tool. (PDF 101 KB)

Additional file 4: Table S2.: Details of sample processing prior to testing with the Xpert MTB/RIF assay. (DOCX 14 KB)

Below are the links to the authors’ original submitted files for images.

Authors’ original file for figure 1

Authors’ original file for figure 2

Authors’ original file for figure 3

Authors’ original file for figure 4

Authors’ original file for figure 5

## References

[1] World Health Organization: Global Tuberculosis Report 2013. World Health Organization, Geneva, 2013.

[2] Perkins M, Cunningham J (2007). Facing the Crisis: Improving the Diagnosis of Tuberculosis in the HIV Era. J Infect Dis.

[3] Golden MP, Vikram HR (2005). Extrapulmonary tuberculosis: an overview. Am Fam Physician.

[4] Chakravorty S, Sen MK, Tyagi JS (2005). Diagnosis of extrapulmonary tuberculosis by smear, culture, and PCR using universal sample processing technology. J Clin Microbiol.

[5] Lawn SD, Mwaba P, Bates M, Piatek A, Alexander H, Marais BJ, Cuevas LE, McHugh TD, Zijenah L, Kapata N, Abubakar I, McNerney R, Hoelscher M, Memish ZA, Migliori GB, Kim P, Maeurer M, Schito M, Zumla A (2013). Advances in tuberculosis diagnostics: The Xpert MTB/RIF assay and future prospects for a point-of-care test. Lancet Infect Dis.

[6] Steingart KR, Sohn H, Schiller I, Kloda LA, Boehme CC, Pai M, Dendukuri N: Xpert (R) MTB/RIF assay for pulmonary tuberculosis and rifampicin resistance in adults (Review). *Cochrane database of systematic reviews* 2013, 1:CD009593., 10.1002/14651858.CD009593.pub2 PMC4470352 23440842

[7] World Health Organization: Policy statement: automated real-time nucleic acid amplification technology for rapid and simultaneous detection of tuberculosis and rifampicin resistance: Xpert MTB/RIF system. World Health Organization, Geneva, 2011. 26158191

[8] WHO monitoring of Xpert MTB/RIF roll-out [http://who.int/tb/laboratory/mtbrifrollout/en/]

[9] Moher D, Liberati A, Tetzlaff J, Altman DG (2009). Preferred reporting items for systematic reviews and meta-analyses: the PRISMA statement. PLoS Med.

[10] Reitsma JB, Glas AS, Rutjes AW, Scholten RJ, Bossuyt PM, Zwinderman AH (2005). Bivariate analysis of sensitivity and specificity produces informative summary measures in diagnostic reviews. J Clin Epidemiol.

[11] Whiting PF, Rutjes AW, Westwood ME, Mallett S, Deeks JJ, Reitsma JB, Leeflang MM, Sterne JA, Bossuyt PM (2011). QUADAS-2: a revised tool for the quality assessment of diagnostic accuracy studies. Ann Intern Med.

[12] Ablanedo-Terrazas Y, Alvarado-Delabarrera C, Hernandez-Juan R, Ruiz-Cruz M, Reyes-Teran G: Xpert MTB/RIF for diagnosis of tuberculous cervical lymphadenitis in HIV-infected patients. *The Laryngoscope* Epub ahead of print 2013, Dec 9., 10.1002/lary.24478 24166585

[13] Al-Ateah SM, Al-Dowaidi MM, El-Khizzi NA (2012). Evaluation of direct detection of Mycobacterium tuberculosis complex in respiratory and non-respiratory clinical specimens using the Cepheid Gene Xpert (R) system. Saudi Med J.

[14] Armand S, Vanhuls P, Delcroix G, Courcol R, Lemaitre N (2011). Comparison of the Xpert MTB/RIF test with an IS6110-TaqMan real-time PCR assay for direct detection of Mycobacterium tuberculosis in respiratory and nonrespiratory specimens. J Clin Microbiol.

[15] Bates M, O'Grady J, Maeurer M, Tembo J, Chilukutu L, Chabala C, Kasonde R, Mulota P, Mzyece J, Chomba M, Mukonda L, Mumba M, Kapata N, Rachow A, Clowes P, Hoelscher M, Mwaba P, Zumla A (2013). Assessment of the Xpert MTB/RIF assay for diagnosis of tuberculosis with gastric lavage aspirates in children in sub-Saharan Africa: a prospective descriptive study. Lancet Infect Dis.

[16] Causse M, Ruiz P, Gutierrez-Aroca JB, Casal M (2011). Comparison of two molecular methods for rapid diagnosis of extrapulmonary tuberculosis. J Clin Microbiol.

[17] Deggim V, Somoskovi A, Voit A, Bottger EC, Bloemberg GV (2013). Integrating the Xpert MTB/RIF Assay into a Diagnostic Workflow for Rapid Detection of Mycobacterium tuberculosis in a Low-Prevalence Area. J Clin Microbiol.

[18] Feasey NA, Banada PP, Howson W, Sloan DJ, Mdolo A, Boehme C, Chipungu GA, Allain TJ, Heyderman RS, Corbett EL, Alland D (2013). Evaluation of Xpert MTB/RIF for Detection of Tuberculosis from Blood Samples of HIV-Infected Adults Confirms Mycobacterium tuberculosis Bacteremia as an Indicator of Poor Prognosis. J Clin Microbiol.

[19] Friedrich SO, von Groote-Bidlingmaier F, Diacon AH (2011). Xpert MTB/RIF assay for diagnosis of pleural tuberculosis. J Clin Microbiol.

[20] Hanif SNM, Eldeen HS, Ahmad S, Mokaddas E (2011). GeneXpert MTB/RIF for rapid detection of Mycobacterium tuberculosis in pulmonary and extra-pulmonary samples. Int J Tubercul Lung Dis.

[21] Hillemann D, Rusch-Gerdes S, Boehme C, Richter E (2011). Rapid molecular detection of extrapulmonary tuberculosis by the automated GeneXpert MTB/RIF system. J Clin Microbiol.

[22] Ioannidis P, Papaventsis D, Karabela S, Nikolaou S, Panagi M, Raftopoulou E, Konstantinidou E, Marinou I, Kanavaki S (2011). Cepheid GeneXpert MTB/RIF assay for Mycobacterium tuberculosis detection and rifampin resistance identification in patients with substantial clinical indications of tuberculosis and smear-negative microscopy results. J Clin Microbiol.

[23] Lawn SD, Kerkhoff AD, Vogt M, Wood R (2012). High diagnostic yield of tuberculosis from screening urine samples from HIV-infected patients with advanced immunodeficiency using the Xpert MTB/RIF assay. J Acquir Immune Defic Syndr.

[24] Ligthelm LJ, Nicol MP, Hoek KG, Jacobson R, van Helden PD, Marais BJ, Warren RM, Wright CA (2011). Xpert MTB/RIF for rapid diagnosis of tuberculous lymphadenitis from fine-needle-aspiration biopsy specimens. J Clin Microbiol.

[25] Malbruny B, Le Marrec G, Courageux K, Leclercq R, Cattoir V (2011). Rapid and efficient detection of Mycobacterium tuberculosis in respiratory and non-respiratory samples. Int J Tubercul Lung Dis.

[26] Miller MB, Popowitch EB, Backlund MG, Ager EP (2011). Performance of Xpert MTB/RIF RUO assay and IS6110 real-time PCR for Mycobacterium tuberculosis detection in clinical samples. J Clin Microbiol.

[27] Moure R, Martin R, Alcaide F (2012). Effectiveness of an integrated real-time PCR method for detection of the Mycobacterium tuberculosis complex in smear-negative extrapulmonary samples in an area of low tuberculosis prevalence. J Clin Microbiol.

[28] Nhu NT, Heemskerk D, Thu DD, Chau TT, Mai NT, Nghia HD, Loc PP, Ha DT, Merson L, Thinh TT, Day J, Chau Nv, Wolbers M, Farrar J, Caws M: Evaluation of Xpert MTB/RIF for the diagnosis of tuberculous meningitis. *Journal of clinical microbiology* 2014, 52:226-233., 10.1128/JCM.01834-13 PMC3911435 24197880

[29] Nhu NTQ, Ha DTM, Anh ND, Thu DDA, Duong TN, Quang ND, Lan NTN, Van Quyet T, Tuyen NTB, Ha VT, Giang DC, Dung NH, Wolbers M, Farrar J, Caws M: Evaluation of Xpert MTB/RIF and MODS assay for the diagnosis of pediatric tuberculosis. *BMC Infect Dis* 2013, 13:31., 10.1186/1471-2334-13-31 PMC3562258 23343418

[30] Nicol MP, Spiers K, Workman L, Isaacs W, Munro J, Black F, Zemanay W, Zar HJ (2013). Xpert MTB/RIF Testing of Stool Samples for the Diagnosis of Pulmonary Tuberculosis in Children. Clin Infect Dis.

[31] Peter JG, Theron G, Muchinga TE, Govender U, Dheda K (2012). The diagnostic accuracy of urine-based Xpert MTB/RIF in HIV-infected hospitalized patients who are smear-negative or sputum scarce. PLoS One [Electron Res].

[32] Porcel JM, Palma R, Valdes L, Bielsa S, San-Jose E, Esquerda A (2013). Xpert MTB/RIF in pleural fluid for the diagnosis of tuberculosis. Int J Tubercul Lung Dis.

[33] Teo J, Jureen R, Chiang D, Chan D, Lin R (2011). Comparison of two nucleic acid amplification assays, the Xpert MTB/RIF assay and the amplified mycobacterium tuberculosis direct assay, for detection of Mycobacterium tuberculosis in respiratory and nonrespiratory specimens. J Clin Microbiol.

[34] Tortoli E, Russo C, Piersimoni C, Mazzola E, Dal Monte P, Pascarella M, Borroni E, Mondo A, Piana F, Scarparo C, Coltella L, Lombardi G, Cirillo DM (2012). Clinical validation of Xpert MTB/RIF for the diagnosis of extrapulmonary tuberculosis. (Eur Respir J.

[35] Vadwai V, Boehme C, Nabeta P, Shetty A, Alland D, Rodrigues C (2011). Xpert MTB/RIF: A new pillar in diagnosis of extrapulmonary tuberculosis?. J Clin Microbiol.

[36] Van Rie A, Page-Shipp L, Mellet K, Scott L, Mkhwnazi M, Jong E, Omar T, Beylis N, Stevens W, Sanne I, Menezes CN: Diagnostic accuracy and effectiveness of the Xpert MTB/RIF assay for the diagnosis of HIV-associated lymph node tuberculosis. *European Journal of Clinical Microbiology and Infectious Diseases* 2013, 32:1409-1415., 10.1007/s10096-013-1890-0 23660698

[37] Zeka AN, Tasbakan S, Cavusoglu C (2011). Evaluation of the GeneXpert MTB/RIF assay for rapid diagnosis of tuberculosis and detection of rifampin resistance in pulmonary and extrapulmonary specimens. J Clin Microbiol.

[38] Zmak L, Jankovic M, Jankovic VK (2013). Evaluation of Xpert MTB/RIF assay for rapid molecular diagnosis of tuberculosis in a two-year period in Croatia. Int J Mycobacteriol.

[39] Patel VB, Theron G, Lenders L, Matinyena B, Connolly C, Singh R, Coovadia Y, Ndung'u T, Dheda K (2013). Diagnostic Accuracy of Quantitative PCR (Xpert MTB/RIF) for Tuberculous Meningitis in a High Burden Setting: A Prospective Study. PLoS Med/Publ Libr Sci.

[40] Christopher DJ, Schumacher SG, Michael JS, Luo R, Balamugesh T, Duraikannan P, Pollock NR, Pai M, Denkinger CM (2013). Performance of Xpert MTB/RIF on pleural tissue for the diagnosis of pleural tuberculosis. Eur Respir J.

[41] Williamson DA, Basu I, Bower J, Freeman JT, Henderson G, Roberts SA (2012). An evaluation of the Xpert MTB/RIF assay and detection of false-positive rifampicin resistance in Mycobacterium tuberculosis. Diagn Microbiol Infect Dis.

[42] Walters E, Gie RP, Hesseling AC, Friedrich SO, Diacon AH, Gie RP (2012). Rapid diagnosis of pediatric intrathoracic tuberculosis from stool samples using the Xpert MTB/RIF Assay: a pilot study. Pediatr Infect Dis J.

[43] World Health Organization: Automated real-time nucleic acid amplification technology for rapid and simultaneous detection of tuberculosis and rifampicin resistance: Xpert MTB/RIF assay for the diagnosis of pulmonary and extra-pulmonary TB in adults and children. Policy update. World Health Organization, Geneva, 2013. 25473701

[44] Denkinger CM, Schumacher SG, Boehme CC, Dendukuri N, Pai M, Steingart KR: Xpert MTB/RIF assay for the diagnosis of extrapulmonary tuberculosis: a systematic review and meta-analysis. *European Respiratory Journal* 2014, 44:435-446., 10.1183/09031936.00007814 24696113

[45] Helb D, Jones M, Story E, Boehme C, Wallace E, Ho K, Kop J, Owens MR, Rodgers R, Banada P, Safi H, Blakemore R, Lan NT, Jones-López EC, Levi M, Burday M, Ayakaka I, Mugerwa RD, McMillan B, Winn-Deen E, Christel L, Dailey P, Perkins MD, Persing DH, Alland D (2010). Rapid detection of Mycobacterium tuberculosis and rifampin resistance by use of on-demand, near-patient technology. J Clin Microbiol.

[46] Marais BJ, Gie RP, Schaaf HS, Hesseling AC, Enarson DA, Beyers N (2006). The spectrum of disease in children treated for tuberculosis in a highly endemic area. Int J Tuberc Lung Dis.

[47] Rock RB, Olin M, Baker CA, Molitor TW, Peterson PK (2008). Central Nervous System Tuberculosis: Pathogenesis and Clinical Aspects. Clin Microbiol Rev.

[48] Katti MK (2004). Pathogenesis, diagnosis, treatment, and outcome aspects of cerebral tuberculosis. Med Sci Monit.

[49] Light RW (2010). Update on tuberculous pleural effusion. Respirology.

[50] Perez-Velez CM, Marais BJ (2012). Tuberculosis in children. N Engl J Med.

[51] Lawn SD, Kerkhoff AD, Burton R, Schutz C, van Wyk G, Vogt M, Pahlana P, Nicol M, Meintjes G: Massive Diagnostic Yield of HIV-Associated Tuberculosis Using Rapid Urine Assays in South Africa. In *Program and abstracts of the 2014 Conference on Retroviruses and Opportunistic Infections*. International AIDS Society (IAS). *March 3–6, 2014; Boston, MA, USA* Abstract 811LB.

[52] Osman M, Simpson JA, Caldwell J, Bosman M, Nicol M (2014). GeneXpert MTB/RIF version G4 for identification of rifampin-resistant tuberculosis in a programmatic setting. J Clin Microbiol.

