# Supplementary material for: Diagnostic accuracy of the Xpert MTB/RIF assay for extrapulmonary and pulmonary tuberculosis when testing non-respiratory samples: a systematic review
Source: BMC Infect Dis. 2014 Dec 31;14:709. doi: 10.1186/s12879-014-0709-7 (PMC4298952; doi:10.1186/s12879-014-0709-7)
Supplement: Supplementary file 1 — Additional file 1: Characteristics of excluded studies. (DOCX 14 KB) [file 12879_2014_709_MOESM1_ESM.docx]

**Additional file 1: Table S1**: Characteristics of excluded studies

| First Author | Year | Age | % Male | % HIV | Study Setting | Sampling Method* | Gold standard | Sample types  (total n specimens analysed) | Sample used for gold standard | Composite reference standard included |
| --- | --- | --- | --- | --- | --- | --- | --- | --- | --- | --- |
| Christopher  India | 2013 | Adults  Median age 46 | 80 | NR | Clinical | Prospective  Consecutive | CRS | Pleural fluid, pleural tissue biopsy (146) | As for Xpert | Smear positive *and/or* culture positive fluid/biopsy *and/or* granulomas on tissue biopsy *and/or* MTB in sputum |
| Patel  South Africa | 2013 | Not all results recorded | NR | 87 | Clinical | Prospective  Consecutive | CRS | CSF (204) | As for Xpert | Culture *and/or* Amplicor *and/or* clinical symptoms and received TB treatment |
| Walters  South Africa | 2012 | Children <14 (Median 17.2 months) | 35 | 8.6 | Clinical | Prospective  Unspecified | Liquid culture | Gastric aspirate and faeces (23) | Gastric aspirate | N/A |
| Williamson  New Zealand | 2012 | NR | NR | NR | Clinical | Prospective  Consecutive | Solid and liquid culture | Tissue/biopsy, Lymph node, Middle ear washings (9) | As for Xpert | N/A |

*Sample selection: Study units selected prospectively, or retrospectively from existing samples; Consecutive, random or convenience sampling method. ‘Unspecified’ refers to studies where there was no clear indication how the study participants were chosen
