# Supplementary figures and images for: Diagnostic accuracy of the Xpert MTB/RIF assay for extrapulmonary and pulmonary tuberculosis when testing non-respiratory samples: a systematic review
Source: BMC Infect Dis. 2014 Dec 31;14:709. doi: 10.1186/s12879-014-0709-7 (PMC4298952; doi:10.1186/s12879-014-0709-7)

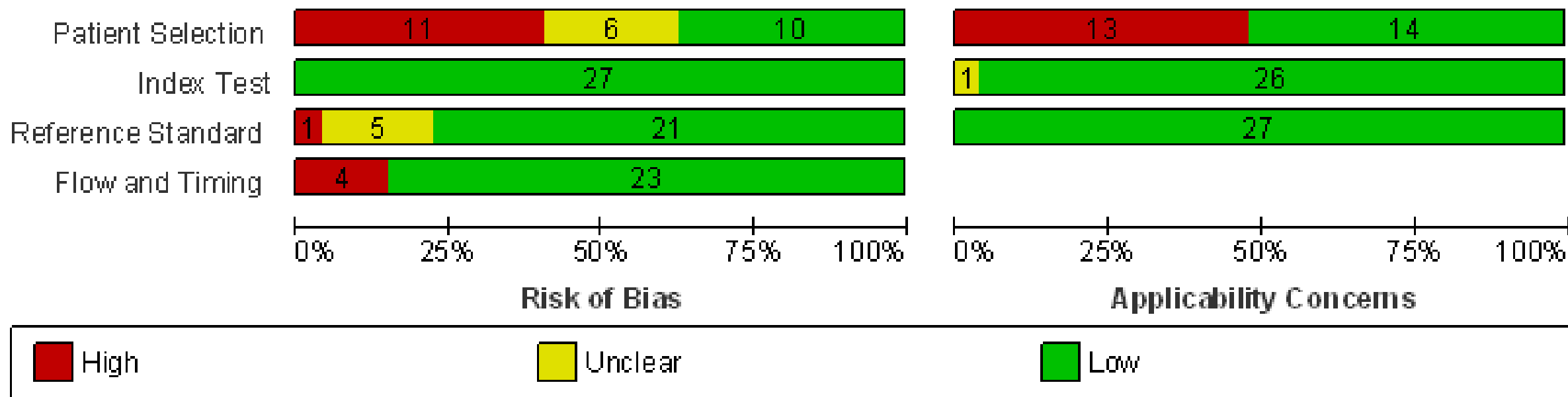

Supplement: Supplementary file 2 — Additional file 2: Figure S1.: Number and percentage of studies within each domain of high, unclear or low risk of bias and concern of applicability, using the QUADAS-2 tool. (PDF 7 KB) [file 12879_2014_709_MOESM2_ESM.pdf]

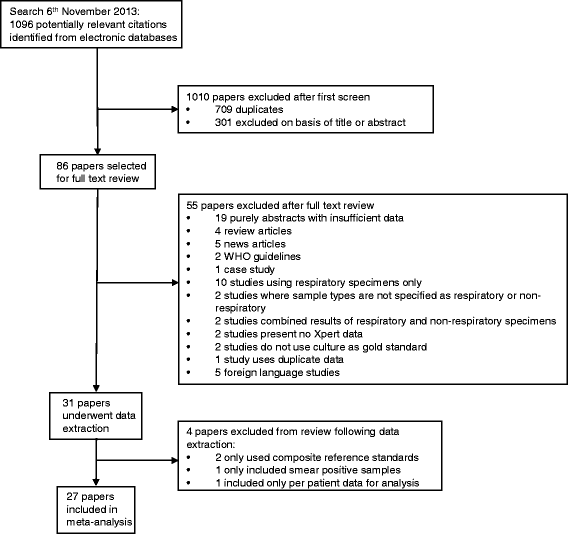

Supplement: Supplementary file 5 — Authors’ original file for figure 1 [file 12879_2014_709_MOESM5_ESM.gif]

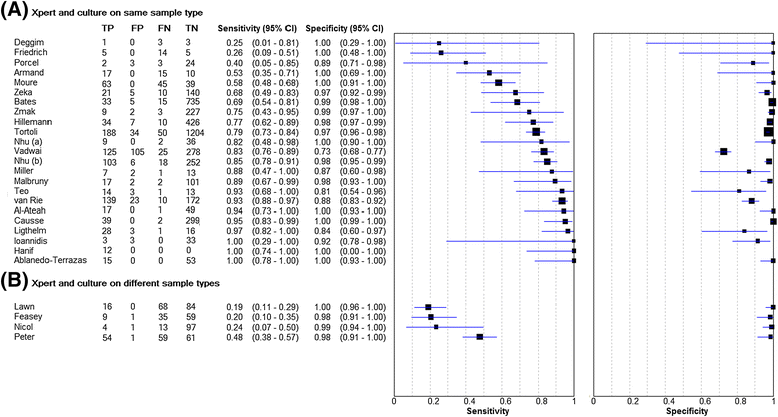

Supplement: Supplementary file 6 — Authors’ original file for figure 2 [file 12879_2014_709_MOESM6_ESM.gif]

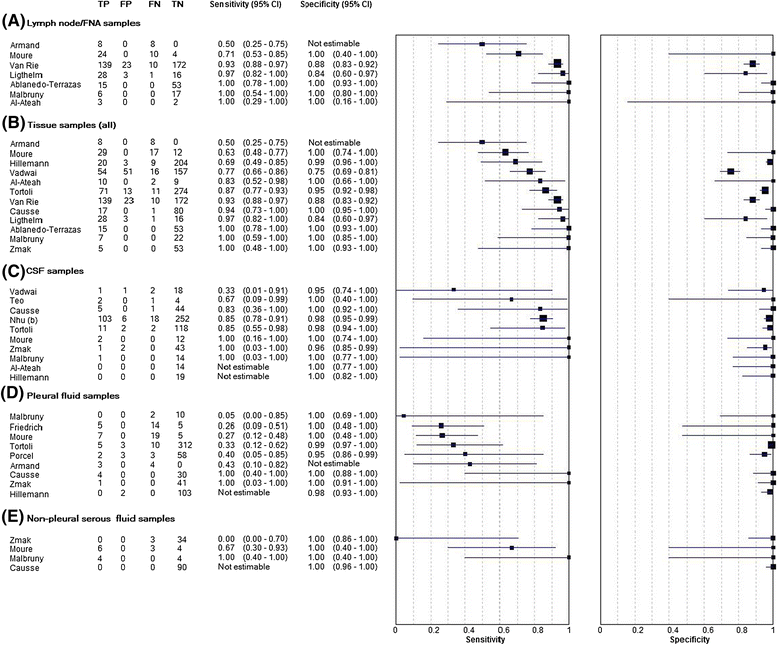

Supplement: Supplementary file 7 — Authors’ original file for figure 3 [file 12879_2014_709_MOESM7_ESM.gif]

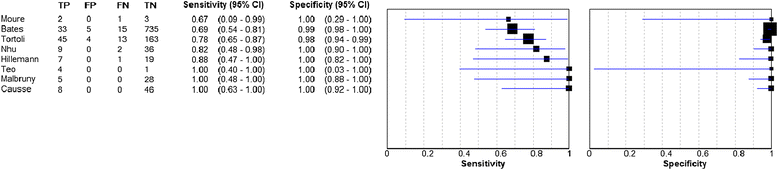

Supplement: Supplementary file 8 — Authors’ original file for figure 4 [file 12879_2014_709_MOESM8_ESM.gif]

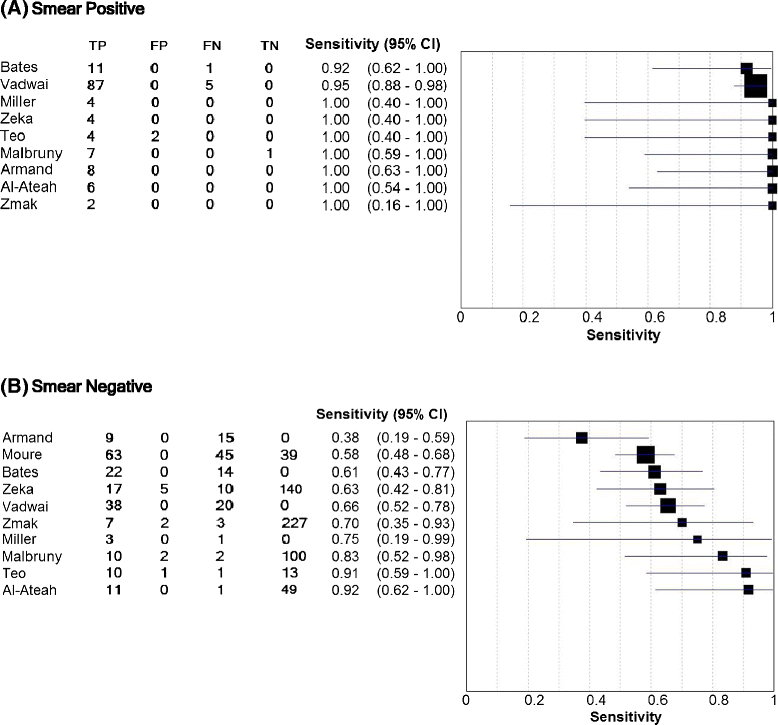

Supplement: Supplementary file 9 — Authors’ original file for figure 5 [file 12879_2014_709_MOESM9_ESM.gif]
