# Supplementary material for: Diagnostic accuracy of the Xpert MTB/RIF assay for extrapulmonary and pulmonary tuberculosis when testing non-respiratory samples: a systematic review
Source: BMC Infect Dis. 2014 Dec 31;14:709. doi: 10.1186/s12879-014-0709-7 (PMC4298952; doi:10.1186/s12879-014-0709-7)
Supplement: Supplementary file 3 — Additional file 3: Figure S2.: Scoring of each study for methodological quality for bias and applicability, using the QUADAS-2 tool. (PDF 101 KB) [file 12879_2014_709_MOESM3_ESM.pdf]

	Risk of Bias				Applicability Concerns		
	Patient Selection	Index Test	Reference Standard	Flow and Timing	Patient Selection	Index Test	Reference Standard
Ablanedo-Terrazas 2013	+	+	+	+	+	+	+
Al-Ateah 2012	+	+	+	+	−	+	+
Armand 2011	−	+	+	+	−	+	+
Bates 2013	+	+	+	+	+	+	+
Causse 2011	+	+	?	+	+	+	+
Deggim 2013	+	+	+	+	−	+	+
Feasey 2013	−	+	+	−	+	+	+
Friedrich 2011	+	+	?	+	+	+	+
Hanif 2011	?	+	+	−	−	+	+
Hillemann 2011	+	+	+	+	−	+	+
Ioannidis 2011	−	+	+	+	−	?	+
Lawn 2012	−	+	+	+	+	+	+
Ligthelm 2011	?	+	+	+	+	+	+
Malbruny 2011	?	+	+	+	−	+	+
Miller 2011	−	+	+	+	−	+	+
Moure 2011	−	+	+	+	−	+	+
Nhu (a) 2013	+	+	+	+	+	+	+
Nhu (b) 2013	+	+	+	+	+	+	+
Nicol 2013	−	+	+	+	+	+	+
Peter 2012	−	+	?	−	+	+	+
Porcel 2013	−	+	−	+	+	+	+
Teo 2011	?	+	?	+	−	+	+
Tortoli 2012	+	+	+	+	−	+	+
Vadwai 2011	−	+	+	−	+	+	+
van Rie 2013	−	+	?	+	+	+	+
Zeka 2011	?	+	+	+	−	+	+
Zmak 2013	?	+	+	+	−	+	+

− High ? Unclear + Low
