# Supplementary material for: Diagnostic accuracy of the Xpert MTB/RIF assay for extrapulmonary and pulmonary tuberculosis when testing non-respiratory samples: a systematic review
Source: BMC Infect Dis. 2014 Dec 31;14:709. doi: 10.1186/s12879-014-0709-7 (PMC4298952; doi:10.1186/s12879-014-0709-7)
Supplement: Supplementary file 4 — Additional file 4: Table S2.: Details of sample processing prior to testing with the Xpert MTB/RIF assay. (DOCX 14 KB) [file 12879_2014_709_MOESM4_ESM.docx]

**Additional file 4: Table S2.** Details of sample processing prior to testing with the Xpert MTB/RIF assay.

| **Study** | **Samples** | **Volume** | **Decontamination or Digestion** | **Centrifuged** | **Reconstituted*** | **Other points*** |
| --- | --- | --- | --- | --- | --- | --- |
| Ablanedo-Terrazas | Lymph node tissue and FNA | Variable | NALC-NaOH | Yes | PBS – volume not stated | 1:2 SR (volumes not stated) |
| Al-Ateah | Variable | Variable | NALC-NaOH for non-sterile only | Yes | 2.5ml PBS | 1ml:2ml SR |
| Armand | Variable | Variable | NALC-NaOH for non-sterile only | Yes |  | Sediment frozen.  500μl:1.5ml SR |
| Bates | Gastric aspirates | Variable | NALC-NaOH | Yes | 2ml PBS | 0.5ml:1.5ml SR |
| Causse | Variable | Variable | NALC-NaOH for non-sterile only | Yes |  | 1ml:2ml SR |
| Deggim | Variable | Variable – made up to 5ml with distilled water | Nil | No |  | 1ml unprocessed: 2ml SR |
| Feasey | Blood | 20ml | Acid-citrate-dextrose solution | 18ml only | 1ml PBS | 1ml:1ml SR |
| Friedrich | Pleural fluid | 50ml |  | Yes | 2ml PBS | 1ml:? SR (‘as per published protocol’) |
| Hanif | Variable | Not stated |  |  |  | ‘According to instructions’ |
| Hillemann | Variable | Variable | NALC-NaOH | Yes | 1 – 1.5ml PBS | >0.5ml reconstituted decontaminated pellet used in 1:3 ratio with SR |
| Ioannidis | Variable | Variable | NALC-NaOH | Yes |  |  |
| Lawn | Urine | 2ml |  | Yes | 0.75ml PBS | ‘According to manufacturer instructions’ |
| Ligthelm | FNA | Variable smear |  | No | 0.7ml PBS | 1:2 SR |
| Malbruny | Variable | Variable | NALC-NaOH for gastric aspirates only | Yes, except CSF |  | ‘500μl aliquot discharged into cartridge’ |
| Miller | Variable | Variable | NALC-NaOH for non-sterile only | Yes | 2ml PBS | 1:3 SR (volume of decontaminated and concentrated specimen not specified) |
| Moure | Variable | Variable | NALC-NaOH for non-sterile only | Yes, only non-sterile | 2ml PBS for non-sterile | 1ml in a ratio to SR ‘in accordance to manufacturer’s protocol’; sterile samples unprocessed |
| Nhu (a) | Gastric aspirates | Variable | NALC-NaOH | Yes | 0.5ml PBS | 0.5ml:1.5ml SR |
| Nhu (b) | CSF | 7ml |  | Yes - Supernatant removed to leave 0.5ml deposit | 200μl resuspended in 300μl PBS | 0.5ml:1.5ml SR |
| Nicol | Stool | 0.15g in 2.4ml PBS, vortexed, 1ml supernatant removed |  | Yes | 1ml PBS | 1ml:2ml SR |
| Peter | Urine | 1ml |  | No |  | 1ml:2ml SR |
| Porcel | Pleural fluid | Variable |  | Yes | 1ml original supernatant | 1ml:2ml SR |
| Teo | Variable | Variable | NALC-NaOH for non-sterile only | Yes | PBS to final volume 2ml | 0.5ml:1mlSR |
| Tortoli | Variable | Variable | NALC-NaOH for non-sterile only | Yes |  | Only samples where >0.5ml concentrated specimen used, in strict accordance with protocol |
| Vadwai | Variable | Variable | NALC-NaOH for non-sterile only | Yes, only non-sterile | Yes – volume not specified | 1:2 SR. Sterile fluids processed directly. CSF, where volumes usually <1ml, made up to 2ml with SR. |
| Van Rie | FNA | Smear into 4ml saline. |  |  |  | 1ml saline:2ml SR |
| Zeka | Variable | Variable | NALC-NaOH for non-sterile only |  |  | 1:3 SR |
| Zmak | Variable | Variable | NALC-NaOH for non-sterile only |  |  | 0.5ml:1.5ml SR |

*PBS = phosphate buffer solution; SR = sample reagent buffer

Where cells are blank, for example in the reconstituted column – there was no mention of the volume of buffer in which the pellet/sediment was resuspended
